# Supplementary material for: Black flies and Onchocerciasis: Knowledge, attitude and practices among inhabitants of Alabameta, Osun State, Southwestern, Nigeria
Source: PLoS Negl Trop Dis. 2022 Apr 21;16(4):e0010320. doi: 10.1371/journal.pntd.0010320 (PMC9022862; doi:10.1371/journal.pntd.0010320)
Supplement: Attachment — Submitted filename: -plosntd revised response to reviewers comments.docx [file pntd.0010320.s001.docx]

Department of Zoology,

Osun State University,

P.M.B 4494,

Osogbo,

Osun State,

Nigeria.

17th August, 2022.

The Editorial Manager,

PLOS Neglected Tropical Diseases,

San Francisco,

California,

United States of America.

Sir,

**REVISED MANUSCRIPT ON BLACK FLIES AND ONCHOCERCIASIS: KNOWLEDGE, ATTITUDE AND PRACTICES AMONG INHABITANTS OF ALABAMETA, OSUN STATE, SOUTHWESTERN, NIGERIA**

Sequel to your constructive comments on the manuscript Black flies and Onchocerciasis: knowledge, attitude and practices among inhabitants of Alabameta, Osun State, Southwestern, Nigeria’, I write to inform you that the authors have responded to the comments raised by the reviewers. Specifically, kindly find below detailed response:

1. **Reviewer #1**: 1st paragraph

-730 kilometer square = 730 km²

**Response**: The issue on using the unit (km^2^) instead of writing in words the area of the study community have been rectified.

2. **Reviewer #2**: The background and rationale for the study are not well articulated. Therefore, although the study is to provide the knowledge attitudes and practices of the study population, it is difficult to know what the hypothesis was being tested. The statistics was to find difference in dynamics and transmission potential of blackflies. This is not possible through a questionnaire survey. One cannot determine transmission potential of vectors without undertaking vector studies.

**Response**: Articulation of the background and rationale of the study and removal of comments on transmission potential which can only be ascertained through vector studies has been corrected.

3. **Reviewer #1**: 1st paragr.

-what means “Respondents ….were higher than those…” ? You probably mean “were represented at a higher proportion…”

-delete “while students made up the rest of the respondents (8%)”

-is there actually a translation for “Amukuru”?

**Response**: The rephrasal of a sentence in paragraph 1 and deletion of a part of another sentence has been modified since the entire manuscript was rehauled and the translation of ‘Amukuru’ provided.

4. **Reviewer #2**: The conclusions do not reflect on the data presented. The study's relevance does not come out clearly in the light of current onchocerciasis elimination goals. One key element of the study is not the knowledge on blackflies but the fact that there has not been any drug distribution in the community and the lack of knowledge of the treatment for the disease. The conclusion should advocate for either onchocerciasis elimination mapping in the area or the start of an alternate treatment strategy if the level of endemicity is already known. However, none of this is mentioned in the manuscript. Also there is less clarity in the report

**Response**: The conclusion has been modified to reflect the data presented.

5. **Reviewer #1**: The acknowledgment is inappropriate. It should not thank any of the co-authors, nor family members or just anyone “who have contributed in one way or the other“. Only if there were specific contributions to be mentioned. On the other hand, was there any field staff to be mentioned here, and what about the community leaders/chiefs who certainly had to agree to the study in their village?

References:
Seem to be carelessly arranged! In the introduction, “WHO, 1994“is cited, but not listed.

**Response**: Family members etc have been from the acknowledgment and inclusion of the community leaders and members for their support. Also, the reference WHO, 1994 which was cited but not listed as been cited and listed as noted.

6. **Reviewer #1**: The paper presents some rarely reported insights of local knowledge “at the end of the road” with regard to oncho and its vectors. Though easily ignored by the expert community, this knowledge can play a crucial role in oncho elimination efforts. Apart from some specific issues listed below, I would just like to point out the following for revision:
   Since an APOC REMO in 2008 is mentioned, was there ever any oncho control interventions in the particular area? MDA? Can you give any prevalence data? Furthermore, this question should be addressed in the discussion. If people were never confronted with MDA or other oncho control activities, how should they know more about the disease? In any case, the conclusion still remains valid that health education needs to be implemented.

**Response**: These observations have been included in the modified manuscript.

7. **Reviewer #2**: Overall i feel the study needs revision to be accepted for publication. There is a need for a completely re-haul of the write up to bring some clarity. The strength of the manuscript is in the information it provides that there are still many areas with endemic onchocerciasis yet with no knowledge of elimination efforts. Unfortunately, the rationale for the study is not well articulated and the write up has many lapses of mixed up information. In the methodology section, one would expect to know what was done before the ethical approval is provided. The results section has no statistics for any of the observations made and finally, the discussion section is poorly written.

There are many statements that needs clarity Examples are: "Alabameta is highly endemic with onchocerciasis with low community microfilarial load" “It is endemic in many tropical countries”.

**Response**: A complete rehaul of the manuscript was made to effect the comments.

Thank you for the comments as we hope that the editor would find the revised manuscript suitable for publication.

Yours faithfully,

Signed,

Busari, Lateef Oluwatoyin.
