# Supplementary material for: Black flies and Onchocerciasis: Knowledge, attitude and practices among inhabitants of Alabameta, Osun State, Southwestern, Nigeria
Source: PLoS Negl Trop Dis. 2022 Apr 21;16(4):e0010320. doi: 10.1371/journal.pntd.0010320 (PMC9022862; doi:10.1371/journal.pntd.0010320)
Supplement: Attachment — Submitted filename: 1Response to reviewers.docx [file pntd.0010320.s002.docx]

Department of Zoology,

Osun State University,

P.M.B 4494,

Osogbo,

Osun State,

Nigeria.

16th August, 2022.

The Editorial Manager,

PLOS Neglected Tropical Diseases,

San Francisco,

California,

United States of America.

Sir,

**REVISED MANUSCRIPT ON BLACK FLIES AND ONCHOCERCIASIS: KNOWLEDGE, ATTITUDE AND PRACTICES AMONG INHABITANTS OF ALABAMETA, OSUN STATE, SOUTHWESTERN, NIGERIA**

The authors gladly received the reviewers’ constructive comments on the manuscript entitled ‘Black flies and Onchocerciasis: knowledge, attitude and practices among inhabitants of Alabameta, Osun State, Southwestern, Nigeria’ and have appropriately responded to the issues. The responses/changes are highlighted (in bold) in the revised manuscript with highlights version.

Kindly find below detailed response:

1. **Comments:** Why was this community chosen for the study? What kind of medical care service, if any, is available in the community? How many people live in the community? How were the 150 citizens chosen to participate in the survey?

**Responses:**

- The intense biting of black flies had been reported in the community. This characteristics informed its selection for this study. (lines 109 – 110).
- The river usually produces rapids along its course which serves as conducive breeding sites for *S. damnosum* (Adewale et al., 1999). (lines 105 – 107)
- The community lacks basic social amenities such hospital/clinic, electricity, pipe borne water, hospitals, good roads, and recreation center. (lines 110 – 112)
- The community population is about 300 of which majority are youths and school aged children.(lines 115 – 116)
- The residents of the community were mobilized for the survey. A total of 150 structured questionnaires were administered to consented community members..(lines 123 – 125)

2. **Comments:** Has the community been previously surveyed for the presence of onchocerciasis? If so, what was the prevalence? If not, why not? Presumably, ivermectin MDA has not been implemented there.

**Responses:**

- Alabameta had been previously surveyed for onchocerciasis with a prevalence of 33.3% (Ojurongbe *et al*., 2015) (lines 108 -1 09)

3. **Comments:** How close is the nearest community in which ivermectin MDA has been carried out? If MDA has not been carried out in the general vicinity, it may explain the lack of knowledge of the citizens.

**Responses:**

- Despite the high prevalence of onchocerciasis in the community, the community (nor neighboring communities) has not been enrolled on annual treatment of Mass Drug Administration. This may be partly due to its location as Alabameta is one of the hard to reach communities in Ife South Local Government of Osun State.(lines 112 – 115)

Thank you for the comments as we hope that the editor would find the revised manuscript suitable for publication.

Yours faithfully,

Signed,

Busari, Lateef Oluwatoyin.
